# CD39 is upregulated during activation of mouse and human T cells and attenuates the immune response to *Listeria monocytogenes*

**DOI:** 10.1371/journal.pone.0197151

**Published:** 2018-05-09

**Authors:** Friederike Raczkowski, Anne Rissiek, Isabell Ricklefs, Kirsten Heiss, Valéa Schumacher, Kira Wundenberg, Friedrich Haag, Friedrich Koch-Nolte, Eva Tolosa, Hans-Willi Mittrücker

**Affiliations:** Institute for Immunology, University Medical Center Hamburg-Eppendorf, Hamburg, Germany; Karolinska Institutet, SWEDEN

## Abstract

The ectoenzymes CD39 and CD73 degrade extracellular ATP to adenosine. ATP is released by stressed or damaged cells and provides pro-inflammatory signals to immune cells through P2 receptors. Adenosine, on the other hand, suppresses immune cells by stimulating P1 receptors. Thus, CD39 and CD73 can shape the quality of immune responses. Here we demonstrate that upregulation of CD39 is a consistent feature of activated conventional CD4^+^ and CD8^+^ T cells. Following stimulation *in vitro*, CD4^+^ and CD8^+^ T cells from human blood gained surface expression of CD39 but displayed only low levels of CD73. Activated human T cells from inflamed joints largely presented with a CD39^+^CD73^—^ phenotype. In line, in spleens of mice with acute *Listeria monocytogenes*, listeria-specific CD4^+^ and CD8^+^ T cells acquired a CD39^+^CD73^—^ phenotype. To test the function of CD39 in control of bacterial infection, CD39-deficient (CD39^-/-^) mice were infected with *L*. *monocytogenes*. CD39^-/-^ mice showed better initial control of *L*. *monocytogenes*, which was associated with enhanced production of inflammatory cytokines. In the late stage of infection, CD39^-/-^ mice accumulated more listeria-specific CD8^+^ T cells in the spleen than wildtype animals suggesting that CD39 attenuates the CD8^+^ T-cell response to infection. In conclusion, our results demonstrate that CD39 is upregulated on conventional CD4^+^ and CD8^+^ T cells at sites of acute infection and inflammation, and that CD39 dampens responses to bacterial infection.

## Introduction

Extracellular adenine nucleotides are important regulators of immune responses [1, 2]. ATP is released by stressed, damaged or dying cells and acts as a damage-associated molecular pattern (DAMP) provoking and enhancing local immune responses. Immune cells use ionotropic P2X receptors and metabotropic P2Y receptors to sense extracellular ATP (eATP) [1]. Through stimulation of P2Y receptors, eATP acts as a chemotactic factor for granulocytes, macrophages and dendritic cells [3–5]. Stimulation of P2X7 on macrophages activates the NLRP3 inflammasome [6]. P2X receptors are also expressed on T-cell subsets, and eATP stimulation of P2X7 can modulate the function of T_h17_ cells, T_r1_ cells and T_reg_ cells [2, 7–10] and cause apoptosis of T cells [10, 11]. In contrast to eATP, extracellular adenosine is mainly considered as an inhibitory signal of the immune system [12]. By engaging P1 receptors, extracellular adenosine dampens the function of a variety of immune cells, including antigen-presenting cells and T cells, thus dampening the immune response [1, 2, 13–20].

CD39 (ectonucleoside triphosphate diphosphohydrolase 1, ENTPD1) is a major hydrolase for eATP. CD39 hydrolyses eATP and eADP to eAMP, which is further degraded to adenosine by CD73 (ecto-5’ nucleotidase, NT5E) [12, 21]. By degrading eATP, CD39 limits inflammatory processes and, in cooperation with CD73, generates extracellular adenosine, which further dampens immune responses. CD39 and CD73 are expressed on hematopoietic and non-hematopoietic cells, and the expression of these ectoenzymes allows cells to shape the quality of immune responses. The immune modulatory function of CD39 and CD73 has been extensively studied in T_reg_ cells [22–24]. Mouse T_reg_ cells express CD39 and CD73 and generate adenosine to suppress immune responses [22]. CD39 is also expressed by subsets of human T_reg_ cells, but these cells mostly fail to co-express CD73 [25, 26]. For conventional T cells (non-T_reg_ cells), CD39 and CD73 have been proposed as markers of activation and exhaustion [27–30]. It is also possible that, similar to T_reg_ cells, conventional T-cell populations use CD39 to reduce eATP in their environment. This would dampen local inflammation but could also directly interfere with eATP-dependent activation and differentiation processes of these cells. Interestingly, T cells from mucosal tissues express relatively high levels of CD39 and CD73 [31]. In the gut, high expression levels of CD39 and CD73 could protect T cells and other intestinal cells from adverse effects of eATP derived from commensal bacteria of the intestinal lumen [23, 31, 32].

In this study, we have analyzed the expression and function of CD39 on conventional mouse and human CD4^+^ and CD8^+^ T cells following activation and during infection of wildtype and CD39-deficient mice with *Listeria monocytogenes* (Lm). Our results demonstrate that a large fraction of conventional CD4^+^ and CD8^+^ T cells acquired a CD39^+^CD73^—^ phenotype upon activation. In addition, CD39^+^ CD4^+^ and CD8^+^ T cell were enriched in the human memory T-cell compartment, and at sites of acute inflammation such as the synovial fluid of inflamed joints. Following listeria-infection of mice, the majority of listeria-specific CD4^+^ and CD8^+^ T cells were CD39^+^ and CD73^—^. CD39^-/-^ mice showed lower listeria titers at early time points of infection but higher frequencies of listeria-specific CD8^+^ T cells at later time points, indicating that CD39 influenced both innate and acquired responses to *L*. *monocytogenes*. In conclusion, our results suggest a role of CD39 in the regulation of conventional CD4^+^ and CD8^+^ T cells.

## Materials and methods

### Mice and *Listeria monocytogenes* infection

CD39^-/-^ mice [33] on the C57BL/6 background were kindly provided by Drs. Holger Eltzschig and Simon Robson. This study was carried out in strict accordance with the state guidelines. The protocol was approved by local ethics committee of the Behörde für Gesundheit und Verbraucherschutz of the City of Hamburg (Permit numbers: 56/12, 81/14). Mice were housed in the animal facility of the University Medical Center Hamburg-Eppendorf under specific pathogen free conditions in individually ventilated cages with standard food and water ad libitum. During infection experiments, mice were controlled daily and mice with signs of severe disease were euthanized with an O_2_/CO_2_ mixture to minimize suffering.

Mice were infected i.v. with the indicated doses of *L*. *monocytogenes* wildtype strain EGD (Lm) or *L*. *monocytogenes* expressing ovalbumin (LmOVA) [34]. Bacterial inocula were controlled by plating serial dilutions on tryptic soy broth (TSB) agar. For determination of bacterial burdens, organs were homogenized in H_2_O, serial dilutions of homogenates were plated on TSB agar and colonies were counted after 24h incubation at 37°C.

### Isolation and stimulation of cells

Cells from mouse spleens were obtained by mashing the organs through cell sieves into PBS, followed by erythrocyte lysis with lysing buffer (155mM NH_4_Cl, 10mM KHCO_3_, 100μM EDTA, pH 7.2). Cells were incubated in RPMI 1640 medium supplemented with 5% FCS, L-glutamine, pyruvate, gentamicin and 2-mercaptoethanol. For the induction of cytokines in T cells, spleen cells were stimulated for 4h at 37°C with 10^-6^ M ovalbumin peptide (OVA_257-264_; SIINFEKL) and 10^−5^ M listeriolysin O peptide (LLO_189-201_; WNEKYAQAYPNVS) (both JPT, Berlin, Germany) or phorbol 12-myristate 13-acetate (PMA, 50ng/ml, Sigma Aldrich, St. Louis, MO) and ionomycin (1μM, Sigma Aldrich). Brefeldin A (10μg/ml, Sigma Aldrich) was added for the last 3.5h of culture to prevent protein secretion. Cytokine expression was determined by intracellular staining and flow cytometry. For the induction of cytokine secretion, spleen cells were incubated for 16h at 37°C with Lm. Supernatants were collected and analyzed for TNF-α and IL-1β content by ELISA (R&D Systems, Minneapolis, MN).

Unless stated otherwise, peripheral blood was obtained from healthy volunteers. Blood and synovial fluid (SF) of patients with juvenile idiopathic arthritis (JIA) were obtained from patients visiting the University Medical Center Hamburg-Eppendorf, the Altona Children’s Hospital or the Medical Center Bad Bramstedt. Joints of patients were punctured for diagnostic or therapeutic reasons. Collection procedures were approved by the local ethics committee (Ethik-Kommission der Ärztekammer Hamburg. PV5139 for samples from healthy donors and PV3746 for samples from JIA patients). Human peripheral blood mononuclear cells (PBMCs) were isolated from venous blood by Biocoll gradient centrifugation (Biochrom, Berlin, Germany). Cells were cultured in human RPMI 1640 medium supplemented with 10% FCS, L-glutamine, penicillin, streptomycin. For activation of T cells, PBMCs (1×10^6^/ml) were cultured with 0.5μg/ml anti-CD3 mAb (clone OKT3, BioLegend, San Diego, CA). Flow cytometric analysis of cells was performed on the indicated days.

### Flow cytometric analysis

For surface staining of human cells, 100μl of whole blood, 0.5–1×10^6^ PBMCs or 1×10^5^ SF cells were incubated with hIgG (Jackson ImmunoResearch Inc., West Grove, PA) to minimize unspecific antibody binding. Fluorochrome-conjugated mAbs were added and cells were incubated for 30min. In case of whole blood staining, this step was followed by erythrocyte lysis (Lysing Solution, BD Biosciences, San Jose, CA). For exclusion of dead cells a fixable dead cell stain (LIVE/DEAD Cell Viability Assay, Live Technologies, Carlsbad, CA) was added. For intracellular staining of Foxp3, PBMCs were surface stained, washed with PBS and then fixed and permeabilized using Foxp3-Transcription Factor Staining Buffer (eBioscience). To minimize unspecific anti-Foxp3 mAb binding, cells were incubated in permeabilization buffer with 1:100 mouse serum for 10min before the addition of anti-Foxp3 mAb. Cells were incubated for 30min at 4°C and then washed with permeabilization buffer.

Mouse cells were incubated with 10μg/ml 2.4G2 (anti-FcγRII/III; BioXCell, West Lebanon, NH) and 1:100 rat serum in PBS to minimize unspecific antibody binding. Staining was performed on ice with fluorochrome-conjugated mAbs (see below). For detection of intracellular proteins, cells were surface stained and then incubated with a fixable dead cell stain (Pacific Orange succinimidyl ester; Life Technologies) to exclude dead cells from analysis. Cells were washed with PBS and fixed for 20min with PBS, 2% paraformaldehyde at room temperature. Thereafter, cells were washed with PBS, 0.2% BSA, permeabilized with PBS, 0.1% BSA, 0.3% saponin (Sigma, Aldrich), and incubated in this buffer with 1:100 rat serum. After 5min, fluorochrome-conjugated antibodies were added. After further 20min on ice, cells were washed with PBS.

Samples were analyzed with a FACS Canto II or LSRFortessa flow cytometer (BD Biosciences). Results were evaluated using the FlowJo software (Treestar, Ashland, OR, USA).

Fluorochrome-conjugated anti-human mAbs: anti-CD3 (clones UCHT1 or OKT3), anti-CD4 (RPA-T4), anti-CD8α (RPA-T8 or HIT8a), anti-CD25 (2A3), anti-CD39 (A1), anti-CD45RA (HI100), anti-CD73 (AD2), anti-CD127 (HCD127), anti-HLA-DR (L243), anti-CCR7 (G043H7) and Fluorochrome-conjugated anti-mouse mAbs: anti-CD3ε (145-2C11), anti-CD4 (RM4-5), anti-CD8α (53–6.7), anti-CD11b (M1/70), anti-CD39 (24DMS1), anti-CD44 (IM7), anti-CD62L (MEL-14), anti-CD73 (eBioTy/11.8), anti-Gr-1 (RB6-8C5), anti-Ly6C (AL-21), anti-IFN-γ (XMG1.2), anti-TNF-α (MP6-XT22) and anti-IL-6 (MP5-20F3). mAbs were purchased from BD Biosciences, BioLegend, or eBioscience. Ovalbumin-specific CD8^+^ T cells were detected with H-2K^b^OVA_257–264_ dextramers (Immudex, Copenhagen, Denmark).

### Statistical analysis

All statistical analysis was performed with Prism software (GraphPad, La Jolla, CA) using tests indicated in figure legends. A p-value of <0.05 was considered significant (P<0.05:*; P<0.01:**; P<0.001:***; ns: not significant P>0.05).

## Results

### Activation of conventional human T cells results in changes of CD73 and CD39 expression

**Fig 1:**
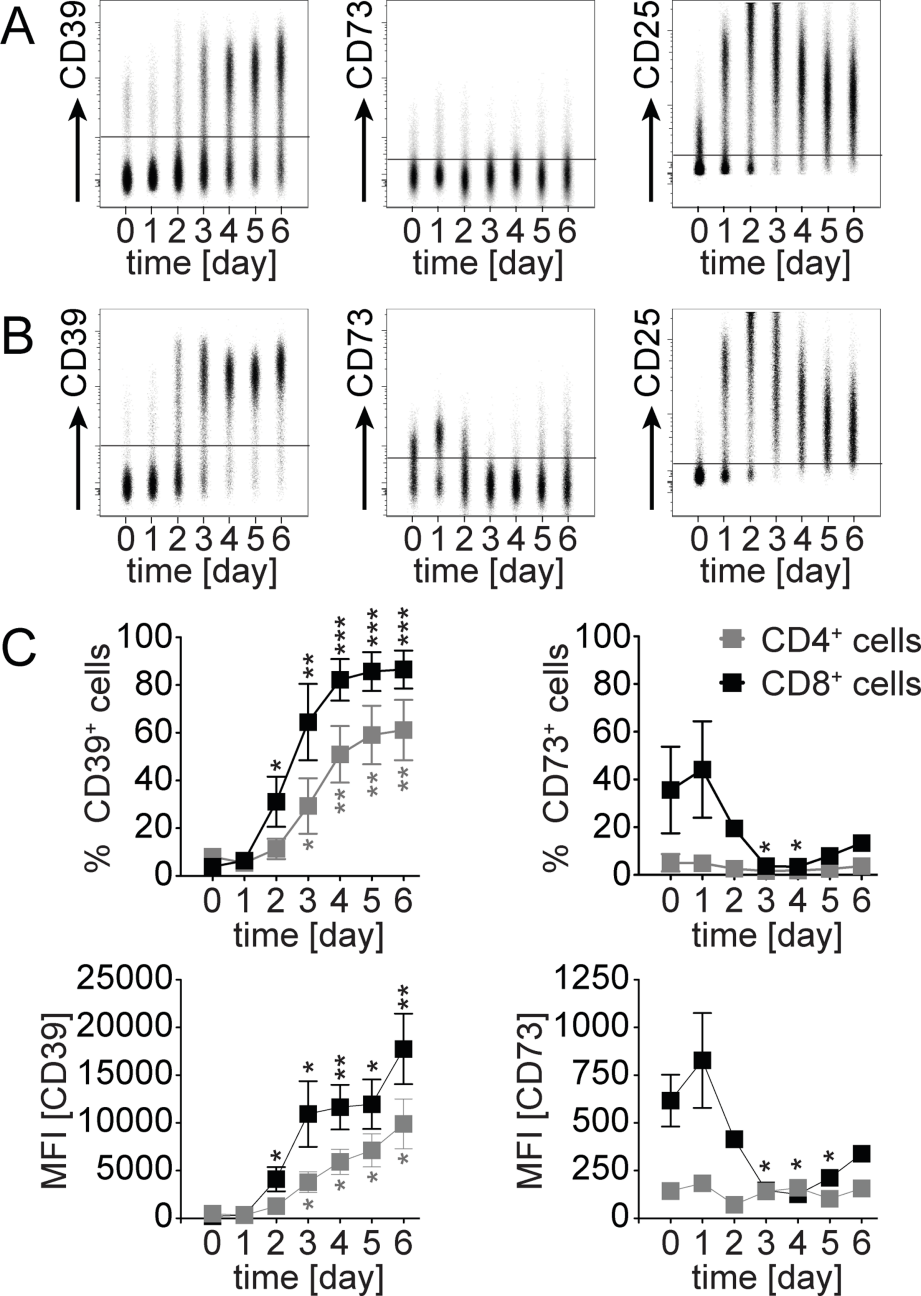
CD39 and CD73 expression on human T cells following activation. PBMCs were stimulated with anti-CD3 mAb. Flow cytometric analysis of CD39, CD73 and CD25 on CD4^+^ and CD8^+^ T cells was performed on days 0–6. Representative concatenated dot plots for surface expression of CD39, CD73 and CD25 on CD4^+^ T cells (A) and CD8^+^ T cells (B) are shown. (C) Changes in percentages (upper panel) and MFI (lower panel) of CD39 and CD73 expression following stimulation. Mean ± SEM of three independent experiments are shown. Unpaired t test, comparison with day 0, * p<0.05, ** p<0.01, *** p<0.001.

The expression and function of CD39 and CD73 has been extensively analyzed on T_reg_ cells [27–29, 35, 36]. However, both proteins are also expressed on subsets of conventional CD4^+^ T cells (non-T_reg_ cells) and CD8^+^ T cells, and the function of CD39 and CD73 on these cells is unclear [27–30] In a first set of experiments, we determined the expression of CD39 and CD73 on the cell surface of human T cells following *in vitro* anti-CD3 mAb stimulation of peripheral blood mononuclear cells (Fig 1). Without stimulation, only small subsets of CD4^+^ T cells from peripheral blood had detectable levels of CD39 or CD73 (Fig 1A and 1C). CD8^+^ T cells were mostly CD39^—^, but a substantial subset of cells was CD73^+^ (Fig 1B and 1C) Upon stimulation, both CD4^+^ and CD8^+^ T cells acquired surface expression of CD39, observed as an increased percentage of CD39^+^ cells as well as mean expression level. At day 4, 50–60% of CD4^+^ T cells and >80% of CD8^+^ T cells were CD39^+^. Compared to CD25 which peaked at around day 2, CD39 expression was delayed by 1–2 days. Expression of CD73 remained low on CD4^+^ T cells after stimulation. Notably, CD8^+^ T cells showed an initial increase in CD73 which then dropped to background level.

**Fig 2:**
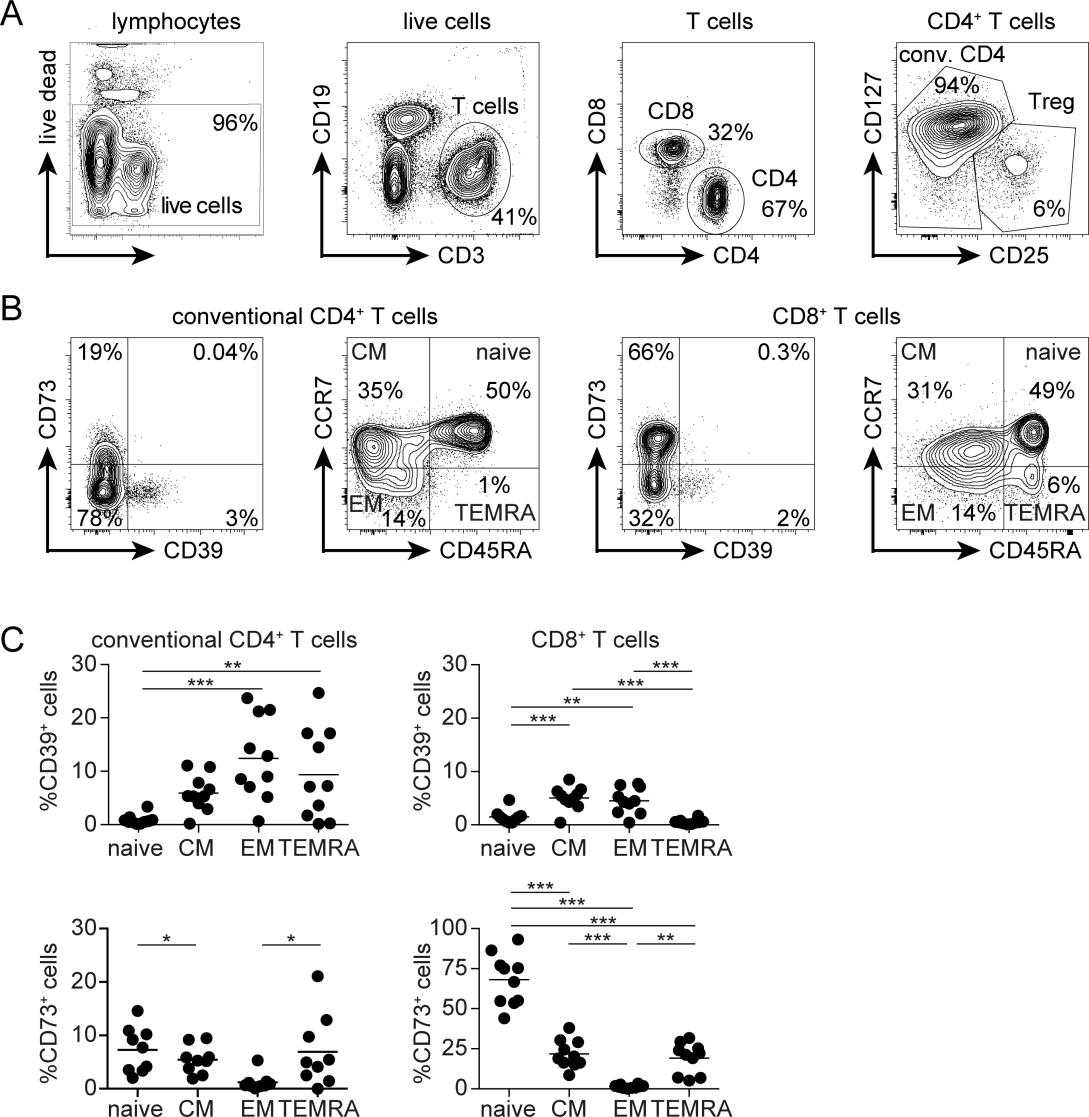
CD39 and CD73 expression profiles of human peripheral blood T-cell subsets. (A) Gating strategy for conventional CD4^+^ and CD8^+^ T cells from human peripheral blood. CD25^+^CD127^—^ T_reg_ cells were excluded. (B) CD39 and CD73 as well as CCR7 and CD45RA expression profile of conventional CD4^+^ and CD8^+^ T cells. Naive: CCR7^+^CD45RA^+^, effector/effector memory (EM): CCR7^—^CD45RA^—^, central memory (CM): CCR7^+^CD45RA^—^ and TEMRA: CCR7^—^CD45RA^+^. (C) Percentages of CD39 and CD73 expression on CD4^+^ and CD8^+^ T-cell subsets from the blood of 10 healthy donors and the means are shown. ANOVA multiple comparison, * p<0.05, ** p<0.01, *** p<0.001.

To correlate CD39 and CD73 expression with the activation and differentiation status of T cells *in vivo*, CD4^+^ and CD8^+^ T cells from peripheral blood of healthy individuals were stained for CD45RA and CCR7 to identify naive (CCR7^+^CD45RA^+^), central memory (CM, CCR7^+^CD45RA^—^), effector/effector memory (EM, CCR7^—^CD45RA^—^) and CD45RA-reverted effector/memory (TEMRA, CCR7^—^CD45RA^+^) T cells and then analyzed for CD39 and CD73 expression (Fig 2). Since T_reg_ cells show the highest expression of CD39 among human T cells [37, 38], they were excluded from the analysis of CD4^+^ T cells (Fig 2A). Naive conventional CD4^+^ T cells and CD8^+^ T cells expressed negligible levels of CD39 (Fig 2C). The other T-cell subsets contained small CD39^+^ populations, with a maximum of 25% CD39^+^ cells for effector memory cells. CD73 was expressed by less than 10% of CD4^+^ T cells at any differentiation stage. In contrast, the majority of naive CD8^+^ T cells was CD73^+^, while most effector and memory CD8^+^ T cells were CD73^—^. CD39^+^CD73^+^ cells were extremely rare in all analyzed populations of conventional CD4^+^ and CD8^+^ T cells from peripheral blood (Fig 2B).

### Accumulation of CD39^+^HLA-DR^+^ T cells in the joints of patients with juvenile arthritis

**Fig 3:**
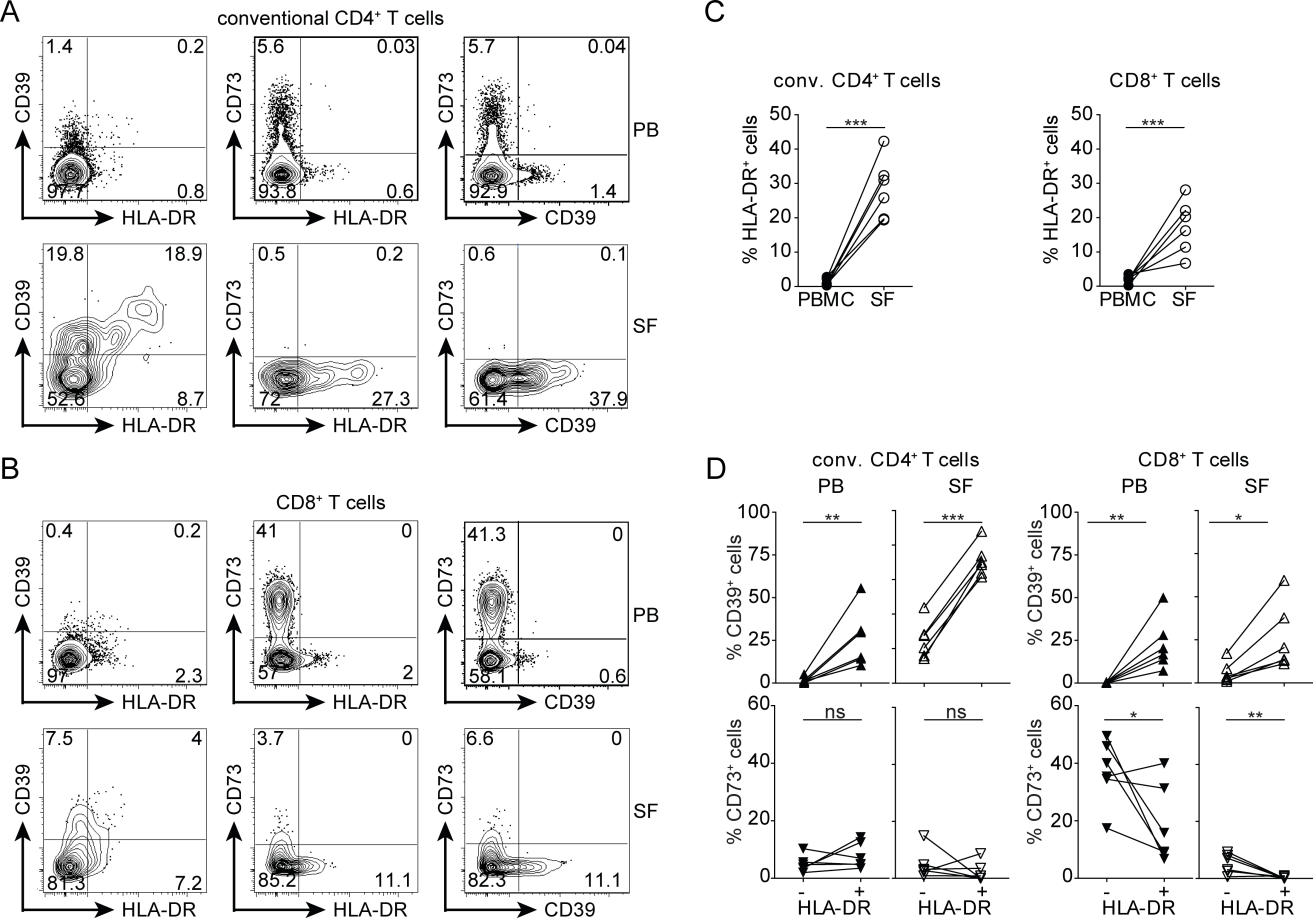
CD39 and CD73 expression on CD4^+^ and CD8^+^ T cells from the synovial fluid of inflamed joints. T cells from the peripheral blood and synovial fluid of JIA patients were analyzed for the expression of HLA-DR, CD39 and CD73. T_reg_ cells were excluded from analysis (see Fig 2A for gating strategy). (A, B) Representative expression profiles for CD39, CD73 and HLA-DR on conventional CD4^+^ T cells (A) and CD8^+^ T cells (B) from peripheral blood (PB) and synovial fluid (SF). (C) Percentages of HLA-DR^+^ cells among CD4^+^ and CD8^+^ T cells in PB and SF. (D) Percentages of CD39^+^ cells (top) and of CD73^+^ cells (bottom) among HLA-DR^+^ and HLA-DR^—^subsets of CD4^+^ and CD8^+^ T cells in peripheral PB and SF. Results for blood and SF of 6 JIA patients are shown. Paired t test, * p<0.05, ** p<0.01, *** p<0.001, ns p>0.05.

To assess the expression of these molecules in an inflammatory setting, we analyzed T cells from a site of chronic inflammation. Mononuclear cells were isolated from peripheral blood (PB) and synovial fluid (SF) of patients with juvenile idiopathic arthritis (JIA). In comparison to the PB, SF contained enlarged subpopulations of activated HLA-DR^+^ cells among CD8^+^ and conventional CD4^+^ T cells (Fig 3). In PB, the relative proportion of CD39^+^ cells was significantly higher among HLA-DR^+^ CD4^+^ and CD8^+^ T cells when compared to HLA-DR^—^cells (Fig 3D). The majority of SF HLA-DR^+^ CD4^+^ T cells expressed CD39 and in this compartment also the HLA-DR^—^ cells had upregulated CD39. Likewise, CD39 expression by SF CD8^+^ T cells was largely restricted to the HLA-DR^+^ subset. CD73 was expressed by few CD4^+^ T cells from both PB and SF, irrespective of their HLA-DR status (Fig 3D). In the PB of most patients, the HLA-DR^+^ CD8^+^ T-cell population contained less CD73^+^ cells than the HLA-DR^—^CD8^+^ T cells. In the SF, only few CD8^+^ T cells expressed CD73 and HLA-DR^+^ CD8^+^ T cells were uniformly CD73^—^. Only marginal frequencies of CD39^+^CD73^+^ conventional CD4^+^ and CD8^+^ T cells were detected in both PB and SF (Fig 3A and 3B). Of note, the CD39 and CD73 expression profile of conventional T cells was similar in the peripheral blood of JIA patients and healthy controls (data not shown).

In conclusion, our results indicate that CD39 and CD73 are dynamically regulated during activation of conventional T cells, and activated CD4^+^ and CD8^+^ T cells at sites of inflammation present a CD39^+^CD73^—^ phenotype.

### Expression of CD39 is upregulated, that of CD73 downregulated by mouse CD4^+^ and CD8^+^ T cells during *L*. *monocytogenes* infection

**Fig 4:**
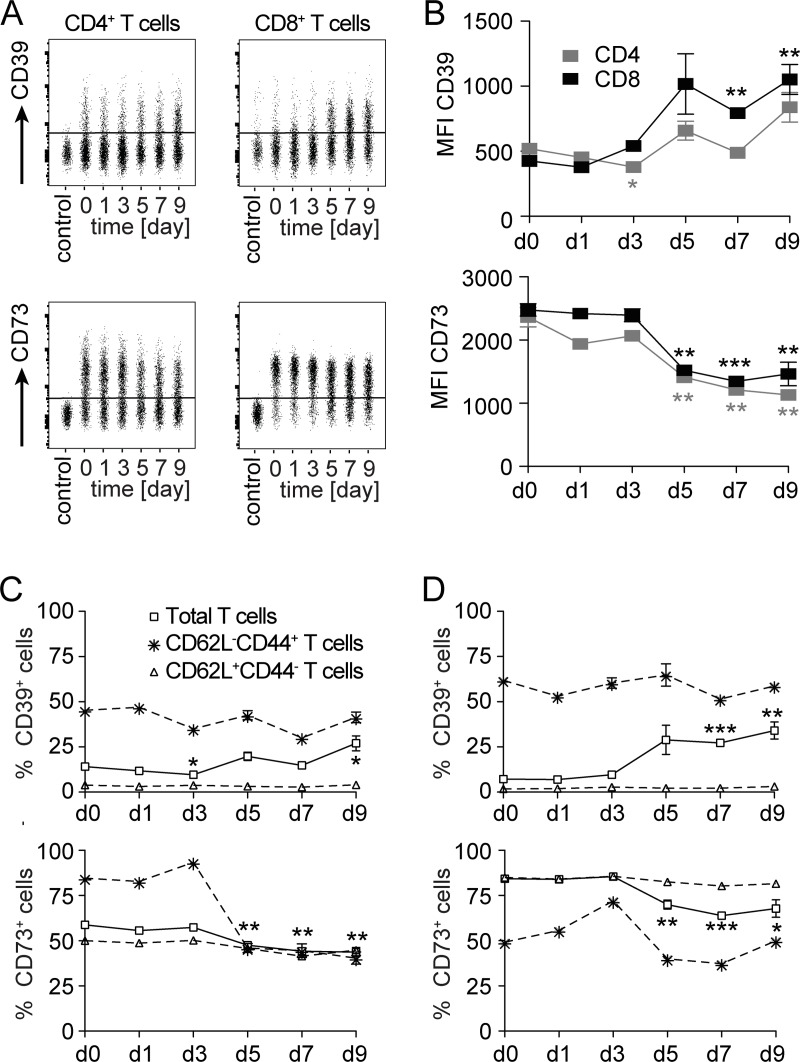
CD39 and CD73 expression profiles of T cells during the course of *L*. *monocytogenes* infection. Wildtype mice were i.v. infected with 1×10^5^ LmOVA. At indicated time points, surface expression of CD39 and CD73 was analyzed on CD4^+^ and CD8^+^ T cells from spleens. (A) Representative concatenated dot plots for CD39 and CD73 expression on CD4 and CD8-gated cells. CD39 staining was controlled with cells from CD39^-/-^ mice, CD73 staining with an isotype control. (B) MFI of CD39 and CD73 staining on CD4 and CD8-gated cells. (C, D) Percentages of CD39^+^ and CD73^+^ cells among CD4^+^ (C) and CD8^+^ T cells (D). Shown are %-values for total, for CD62L^+^CD44^—^ and for CD62L^—^CD44^+^ T-cell populations (see S1 Fig for gating strategy and cell numbers). Values in B-D give the mean ± SEM for three independently analyzed mice per time point and are representative for three independent experiments. Unpaired t test, comparison with day 0 (in C and D only shown for total T-cell populations), * p<0.05, ** p<0.01, *** p<0.001.

To characterize the expression of CD39 and CD73 during an immune response *in vivo*, we infected mice with *L*. *monocytogenes* (Lm). In this infection model, bacterial control depends on the formation of listeria-specific CD4^+^ and CD8^+^ T cells. For both T-cell subsets, immune dominant peptides are either endogenously present or can be recombinantly expressed in listeria, and allow the analysis of T cells responding to the bacterial infection. In our study, mice were infected with an ovalbumin recombinant Lm strain (LmOVA) that allows detection of ovalbumin-specific CD8^+^ T cells. In naive mice, only small subpopulations of CD4^+^ and CD8^+^ T cells expressed CD39 (Figs 4 and S1; d0). Closer analysis revealed that CD39 expression was largely confined to T cells with an activated CD62L^—^CD44^+^ phenotype (Figs 4 and S1). Naive CD4^+^ and CD8^+^ T cells (CD62L^+^CD44^—^) expressed only marginal levels of CD39 (Fig 4). Five days post-infection, percentages and numbers of CD39^+^ CD4^+^ and CD8^+^ T cells as well as the mean CD39 expression level on CD4^+^ and CD8^+^ T cells were increased (Figs 4 and S1). This increase correlated with an expansion of the activated CD62L^—^CD44^+^ T-cell population (S1 Fig). In the uninfected mouse, CD73 was expressed on a large subpopulation of CD4^+^ T cells and on the majority of CD8^+^ T cells (Fig 4). Following infection, CD73 expression in terms of, MFI and frequency of positive cells, decreased on both CD4^+^ and CD8^+^ T cells. Particularly, activated CD62L^—^CD44^+^ T cells showed low levels of CD73 during infection (Fig 4). Due to the overall expansion of T cells during infection, total numbers of CD73^+^ T cells remained stable (S1 Fig). Interestingly, the expansion of the CD62L^—^CD44^+^ CD8^+^ T-cell population was accompanied by an increase in the numbers of CD73^+^ cells in this population.

**Fig 5:**
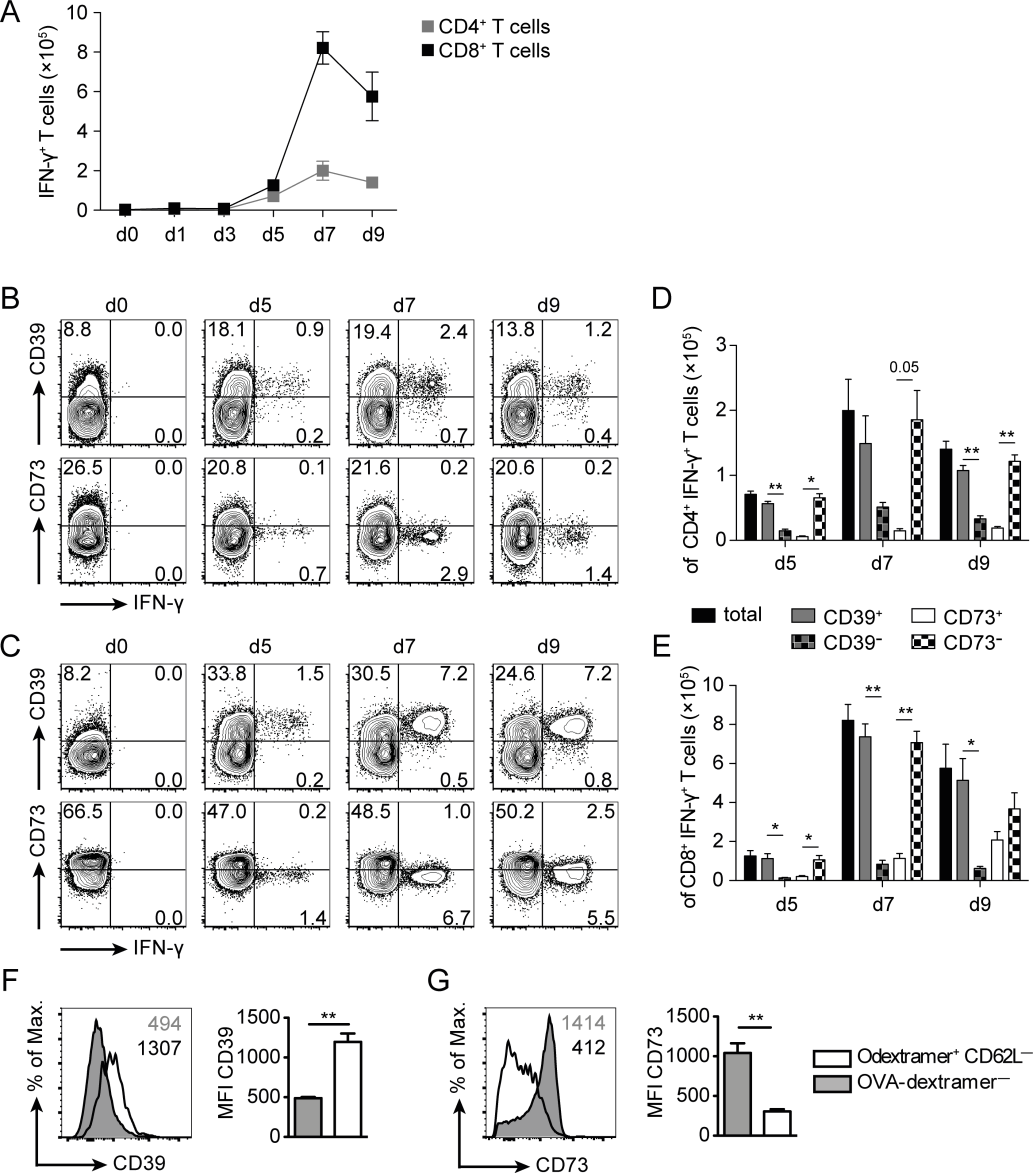
CD39 and CD73 expression profiles of listeria-specific T cells. Wildtype mice were i.v. infected with 1×10^5^ LmOVA. (A-E) Spleens cells were isolated at the indicated time points and stimulated with LLO_189-201_ and OVA_257-264_ peptides for 4h, and then analyzed for expression IFN-γ and CD39 or CD73. Responding T cells were identified by intracellular IFN-γ staining. (A) Numbers of CD4^+^ and CD8^+^ T cells per spleen responding to peptide stimulation with IFN-γ production. Representative FACS plots of CD4 (B) and CD8-gated cells (C). Numbers give the %-values for quadrants. Numbers of total, CD39^+^, CD39^—^, CD73^+^, and CD73^—^cells for IFN-γ^+^ CD4^+^ (D) and for IFN-γ^+^ CD8^+^ T cells (E). Bars give the mean ± SEM. Representative results of CD39 (F) and CD73 expression (G) on OVA_257-264_-dextramer^+^ CD8^+^ T cells at d9 post infection. Open histograms show dextramer^+^ and filled histograms dextramer^—^CD8^+^ T cells. Numbers give the MFI. Bars give the mean ± SEM. Results are representative for at least two independent experiments with three or five individually analyzed mice per group and time point. Paired t test, * p<0.05, ** p<0.01.

To analyze CD39 and CD73 expression on listeria-specific T cells generated during LmOVA infection, spleen cells of infected mice were stimulated with the peptides LLO_189-201_ and OVA_257-264_ to identify specific CD4^+^ and CD8^+^ T cells, respectively [39]. LmOVA infection induced a strong formation of LLO_189-201_–specific CD4^+^ and OVA_257-264_–specific CD8^+^ T cells in spleens of mice (Fig 5A). Expression of CD39 and CD73 was analyzed on IFN-γ^+^ T cells (Fig 5B–5E). Almost all IFN-γ^+^ CD4^+^ and CD8^+^ T cells expressed CD39, while the majority of these cells were negative for CD73. For CD8^+^ T cells, this result could be confirmed on *ex vivo* stained OVA_257-264_-dextramer^+^ CD8^+^ T cells, which exhibited higher levels of CD39 than dextramer-negative CD8^+^ T cells (Fig 5F). CD73 showed an inverse expression profile with reduced expression on dextramer^+^ CD8^+^ T cells and high expression on most of the dextramer-negative cells (Fig 5G). The findings from the dextramer study corroborated results from the study depicted in Fig 5C and 5E and also argue against significant up-regulation of CD39 or down-regulation of CD73 during short-term *in vitro* peptide stimulation. In conclusion, our results indicate that, similar to our observations in humans, CD39 is upregulated and CD73 downregulated on CD4^+^ T_H1_ and CD8^+^ T effector cells generated in response to Lm infection.

### Absence of CD39 enhances early control of *L*. *monocytogenes* infection

**Fig 6:**
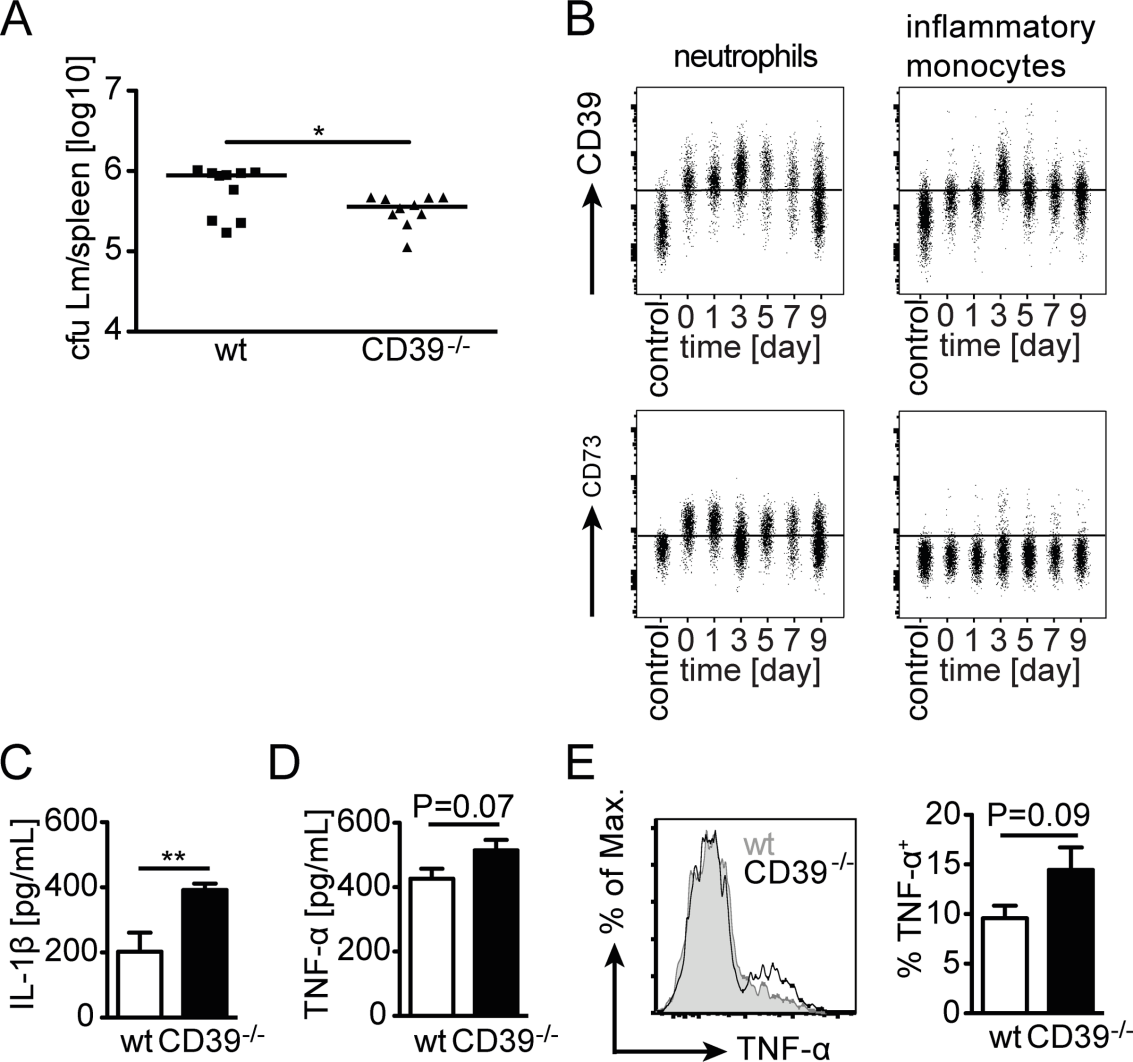
Innate response of CD39^-/-^ mice against *L*. *monocytogenes*. Wildtype and CD39^-/-^ mice were i.v. infected with 5×10^3^ Lm. (A) Bacterial burden in spleens was determined at d2 post infection. Combined results for 10 individually analyzed mice from two independent experiments and the median are shown. cfu, colony forming units. Mann Whitney test, * p<0.05. (B) Wildtype mice were infected with 1×10^5^ LmOVA. At the indicated time points, neutrophils and inflammatory monocytes from the spleen were analyzed for the expression of CD39 and CD73 by flow cytometry. Representative concatenated dot plots for surface expression of CD39 and CD73 on neutrophils and inflammatory monocytes are shown. (See S2 Fig for mean values for groups of mice.) (C, D) Spleen cells from wildtype and CD39^-/-^ mice were cultured with Lm overnight. Supernatants were collected and the concentration of IL-1β (C) and TNF-α (D) was determined by ELISA. Bars present the mean ± SEM of eight values. (E) Wildtype and CD39^-/-^ mice were i.v. infected with 5×10^3^ Lm. On day 2 post infection, spleen cells were isolated and expression of TNF-α was directly analyzed in inflammatory monocytes by intracellular cytokine staining and flow cytometry. Representative histograms and frequency of TNF-α^+^ inflammatory monocytes are given. Bars present the mean ± SEM of five individually analyzed mice. Results are representative for two independent experiments. Unpaired t test, ** p<0.01.

To test whether CD39 participates in the control of Lm, wildtype and CD39^-/-^ mice were infected with Lm and the bacterial burden in spleen was determined on day 2 post infection. Interestingly, CD39^-/-^ mice showed reduced bacterial titers 2 days post-infection (Fig 6A). We detected a median of 8.8×10^5^ and of 3.6×10^5^ listeria in spleens infected wildtype and of CD39^-/-^ mice, respectively (p<0.05). Early control of Lm is facilitated by neutrophils and particularly by inflammatory monocytes recruited to sites of infection. Neutrophils (CD11b^high^ Ly6C^int^ Gr-1^high^ cells) from spleens of naive mice showed surface expression of CD39 and CD73 (Figs 6B and S2). Following Lm infection, expression of CD39 on these cells increased whereas CD73 expression remained at the level observed in naive mice. Inflammatory monocytes (CD11b^high^ Ly6C^high^ Gr-1^int^) from spleens of naive mice expressed CD39 but were negative for CD73. Surface expression of CD39 increased at day 3 of infection, the time point of maximal accumulation of these cells in Lm infected mice [40], and then slowly declined to baseline levels (Figs 6B and S2). Inflammatory monocytes remained CD73^—^ during infection. Accumulation of neutrophils and inflammatory monocytes was determined in spleens of wildtype and CD39^-/-^ mice two days post-infection (S3A and S3B Fig). Although, numbers of both cell populations were reduced in infected CD39^-/-^ mice, reduction did not reach a significant level.

The innate response to Lm is characterized by a substantial production of pro-inflammatory cytokines such as IL-1β, TNF-α and IL-6, which are important for the control of bacteria [41]. To examine whether the lack of CD39 influences the production of these cytokines, spleen cells from wildtype and CD39^-/-^ mice were cultured overnight with Lm, and TNF-α and IL-1β in supernatants were determined (Fig 6C and 6D). Supernatants of wildtype and CD39^-/-^ spleen cells showed robust secretion of IL-1β and TNF-α. Interestingly, CD39^-/-^ spleen cells produced higher levels of IL-1β compared to wildtype spleen cells. For TNF-α, there was only a marginally higher production by CD39^-/-^ cells. Production of TNF-α was also determined by intracellular cytokine staining in inflammatory monocytes from spleens of mice infected for two days with Lm. We observed a trend towards enhanced TNF-α production in cells from CD39^-/-^ mice (Fig 6E). In contrast, neutrophils from wildtype and CD39^-/-^ mice showed similar TNF-α production (S3C Fig). Frequencies of IL-6 producing inflammatory monocytes and neutrophils were comparable between infected wildtype and CD39^-/-^ mice (S3D and S3E Fig).

### Absence of CD39 causes enhanced frequencies of listeria-specific CD8^+^ T cells

**Fig 7:**
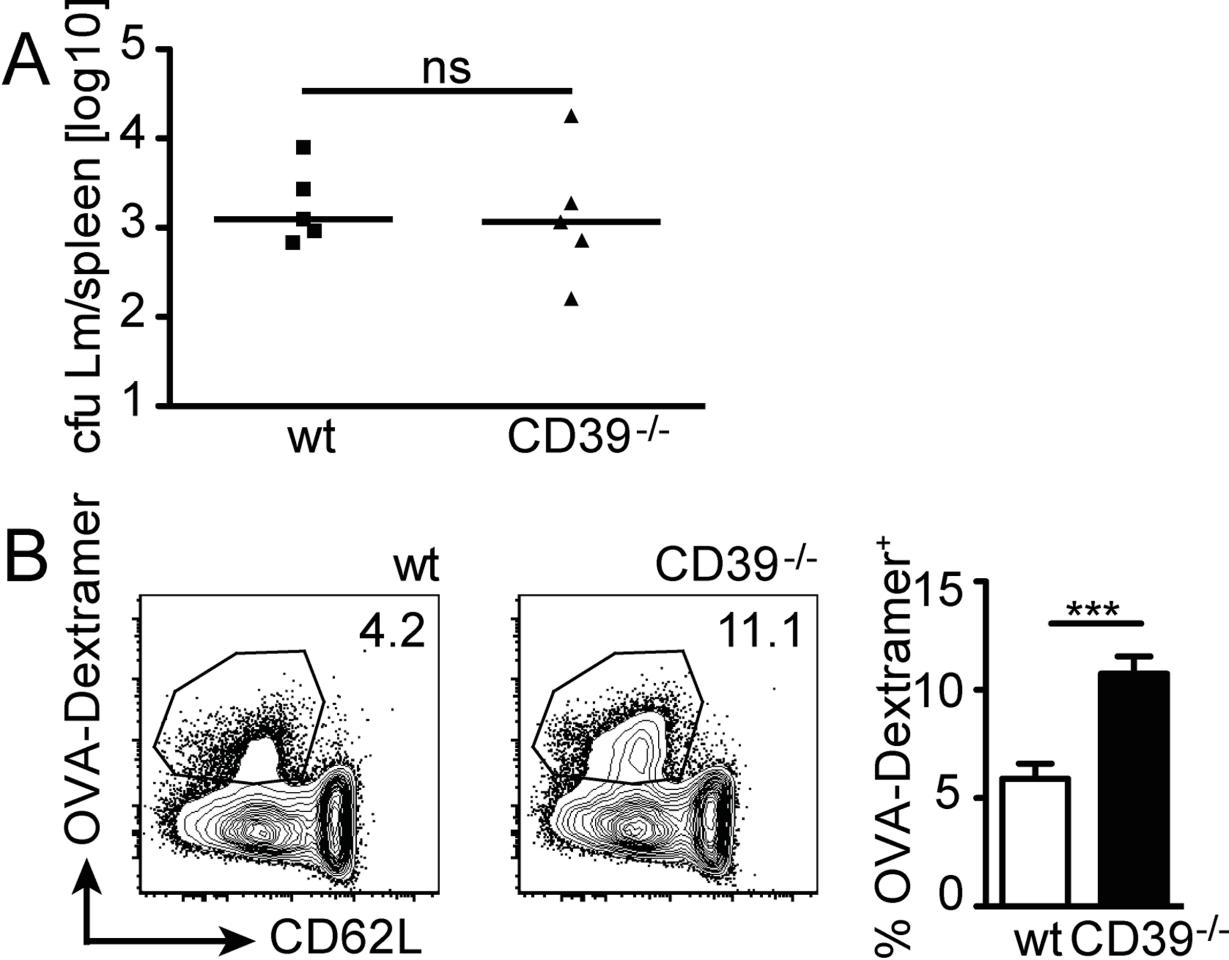
Acquired immune response of CD39^-/-^ mice against *L*. *monocytogenes*. Wildtype and CD39^-/-^ mice were i.v. infected with 5×10^3^ Lm. (A) Bacterial burden in spleens was determined at d7 post infection. Combined results for five individually analyzed mice and the median are shown. cfu, colony forming units. Mann Whitney test, ns p>0.05. (B) Wildtype and CD39^-/-^ mice were i.v. infected with 1×10^5^ LmOVA. On day 9 post infection, CD8^+^ T cells were analyzed with OVA_257-264_-dextramers. Representative dot plots and frequencies of dextramer^+^ cells among CD8^+^ T cells. Bars give the mean ± SEM of seven individually analyzed mice. Combined data of two experiments. Unpaired t test, *** p<0.001.

In the late phase of infection, control of Lm depends on T cells, particularly on CD8^+^ T cells. Wildtype and CD39^-/-^ mice were infected with Lm and bacterial titers in spleens were determined on day 7 post infection. Bacterial titers in wildtype and CD39^-/-^ mice were at similarly low levels, indicating that control of Lm at this stage of response did not depend on CD39 (Fig 7A). To determine the magnitude of Lm-specific T-cell responses, wildtype and CD39^-/-^ mice were infected with LmOVA and CD8^+^ T cells from spleens of infected mice were analyzed for expression of OVA-specific TCRs using OVA_257-264_-dextramers on day 9 post infection. CD39^-/-^ mice had a higher frequency of dextramer^+^ CD8^+^ T cells than wildtype controls (Fig 7B). These results demonstrate that absence of CD39 did not impair the formation of specific T-cell responses against Lm but rather resulted in even stronger accumulation of listeria-specific CD8^+^ T cells.

## Discussion

CD39 and CD73 have been extensively studied on T_reg_ cells in mouse and human [22–24]. Here we focused on the expression of CD39 and CD73 on the cell surface of conventional T cells. Naive human conventional CD4^+^ and CD8^+^ T cells were CD39^—^CD73^—^ and CD39^—^CD73^+^, respectively. Following *in vitro* activation, the majority of T cells acquired a CD39^+^CD73^—^ phenotype. Consistently, circulating human T cells with effector and memory phenotypes were CD73^—^ and contained subsets of CD39^+^ cells while the majority of activated HLA-DR^+^ CD4^+^ T cells in the joints of JIA patients presented with a CD39^+^CD73^—^ phenotype. Observations from the mouse *L*. *monocytogenes* infection model were in line with the results obtained with human cells *in vitro*. During acute infection, listeria-specific CD4^+^ T_H1_ and CD8^+^ T cells predominantly displayed a CD39^+^CD73^—^ phenotype. Our results extend prior studies on human T cells from the peripheral blood and inflammatory sites [24, 27, 30, 35, 36, 38, 42–44] as well as studies in mouse disease models [30, 31, 45–48] in which CD39 upregulation and low CD73 expression was observed on different subsets of activated and memory CD4^+^ and CD8^+^ T cells. Gupta *et al*. demonstrated high CD39 expression on CD8^+^ T cells responding to HCV or HIV infection in humans or to chronic LCMV infection in a mouse model [30]. Interestingly, these cells showed co-expression of PD-1 and presented phenotypical and functional attributes of exhausted CD8^+^ T cells. So far, we have no indication that CD39 expression is limited to exhausted T cells, since CD39^+^ T cells are activated in the synovial fluid and are able to produce inflammatory cytokines in response to acute listeria infection. However, it is well possible that exhausted T cells further upregulate CD39, which would be concordant with the results presented by Gupta *et al*. Two previous studies also observed accumulation of Foxp3^—^CD39^+^ CD4^+^ and CD8^+^ T cells and of CD73^—^CD8^+^ T cells in the synovia of JIA patients [27, 43]. These results are consistent with our observation of accumulation of activated HLA-DR^+^ T cells with a CD39^+^CD73^—^ phenotype at this site of active inflammation. Overall, these results indicate that the CD39^+^CD73^—^ phenotype is a common feature of activated conventional CD4^+^ and CD8^+^ T cells, particularly of T cells engaged in T-cell responses.

In an inflammatory setting, CD39 is substantially increased on conventional T cells while CD73 is largely absent. The expression of CD39 on activated T cells is remarkable because CD39 has so far been mainly linked to immune suppressive processes. CD39 on conventional CD4^+^ and CD8^+^ T cells is enzymatically active and can hydrolyze eATP and eADP to eAMP [27, 30, 36, 42, 49]. CD73 expression on cells in the environment or soluble forms of CD73 could further degrade eAMP to adenosine. Thus, by limiting the availability of eATP and providing eAMP for the formation of adenosine, activated CD39^+^ conventional T cells could mitigate an inflammatory environment. It has also been suggested that ATP released by activated T cells binds P2X receptors on the same cells and thereby supports TCR signaling [2, 7–9]. CD39 on the surface of T cells could block this autocrine loop and interfere with T-cell activation, preventing uncontrolled inflammation.

CD39^-/-^ mice harbor lower listeria titers at day 2 post-infection, suggesting that CD39 interferes with the innate control of bacteria. In addition, we detected enhanced IL-1β production by CD39^-/-^ spleen cells after *in vitro* stimulation with listeria. During the first days of listeria infection, recruitment and activation of neutrophils and particularly of inflammatory monocytes is crucial for limiting bacterial replication. Both cell populations were CD39^+^ and increased CD39 surface expression during infection. Thus, absence of CD39 on these cells might directly be responsible for the enhanced bacterial control. However, absence of CD39 on other cells, such as T_reg_ cells, could likewise improve the innate anti-Lm response. Higher eATP concentrations in the absence of CD39 could boost recruitment of neutrophils and inflammatory monocytes by P2Y receptor mediated mechanisms [3], and result in augmented inflammasome activation and IL-1β secretion via P2X7 stimulation of these cells [50–52]. Deficiency of CD39 might further limit the production of immune suppressive adenosine by CD73. In line with these concepts, CD39^-/-^ and CD73^-/-^ mice present with enhanced local or systemic inflammation and increased levels of inflammatory cytokines in infection and inflammation models resulting in severe, even fatal immunopathology [24, 50, 53–58]. Lack of CD73 has also been associated with improved control of *Salmonella typhimurium* and *Helicobacter felis* infection [24, 53, 56]. Similarly, enhanced inflammation with less restricted responses of inflammatory macrophages and granulocytes could lead to augmented early control of Lm in CD39^-/-^ mice.

Impairment of the CD39-CD73 axis might also explain the enhanced CD8^+^ T-cell response in Lm-infected CD39^-/-^ mice. Such a mechanism has been proposed for the aggravated disease observed in CD73^-/-^ mice in a graft versus host disease model [59] and enhanced anti-graft responses of CD73^-/-^ mice in transplantation models [60, 61]. On the other hand, CD73^-*/-*^ mice show unaltered CD8^+^ T-cell responses to infection with murine cytomegalovirus or recombinant adenovirus [45], and CD39^-/-^ mice fail to respond in an allergic contact hypersensitivity model [55]. Thus, the function of the CD39-CD73 axis depends on the type of adaptive response. The CD39-CD73 axis might also affect CD8^+^ T cell effector functions, in addition to its impact on the frequencies, and thereby regulate the CD8^+^ T cell response. However, so far we have no evidence that the absence of CD39 causes alterations in cytokine production or cytotoxicity of CD8^+^ T cells in the Lm infection. Currently, it is unclear how CD39 attenuates CD8^+^ T-cell responses during Lm-infection. CD39 is expressed on listeria-reactive CD8^+^ T cells and therefore might directly interfere with the activation and function of these cells. Further, T_reg_ cells could utilize CD39 to dampen the anti-listeria T-cell response. CD39 also contributes to the suppressive function of T_r1_ cells [62], which likewise might interfere with the CD8^+^ T-cell response during Lm infection. Finally, we cannot exclude that CD39 expression on other cells in secondary lymphoid tissue or at sites of infection impairs CD8^+^ T-cell priming and function.

In conclusion, we demonstrate that in mouse and human, CD39 expression is not limited to T_reg_ cells but a common feature of conventional CD4^+^ and CD8^+^ T cells activated *in vitro* and *in vivo*. In the Lm infection model, CD39 attenuates both innate and acquired responses. Although the precise mechanism of CD39-mediated suppression still needs to be delineated, our results underscore CD39 as an attractive target for modulating immune responses in autoimmunity and infection.

## Supporting information

S1 Fig CD62L and CD44 expression profiles of T cells during the course of *L*. *monocytogenes* infection. Wildtype mice were i.v. infected with 1×10^5^ LmOVA. At different time points post infection, spleen cells were analyzed by flow cytometry and changes in expression profile as well as percentages and numbers of different T-cell subsets were determined. (A) Representative dot plots for CD4 and CD8 gated cells. (B) Percentages of naive CD62L^+^CD44^—^ and activated CD62L^—^CD44^+^ CD4^+^ and CD8^+^ T cells. Values give the mean ± SEM for 3 independently analyzed mice per time point and are representative for 3 independent experiments. (C, D) Numbers of total, CD62L^+^CD44^—^, and CD62L^—^CD44^+^ cells for CD39^+^ (C) and CD73^+^ (D) CD4^+^ and CD8^+^ T cells. Values give the mean ± SEM for 6 mice per group pooled from 2 independent experiments. Unpaired t test, comparison with day 0 (only shown for total T-cell populations), * p<0.05, ** p<0.01.
(PDF)

S2 Fig Expression of CD39 and CD73 on neutrophils and inflammatory monocytes. (A) Gating strategy: Neutrophile granulocytes were defined as CD11b^high^ Ly6C^int^ Gr-1^high^ and inflammatory monocytes as CD11b^high^ Ly6C^high^ Gr-1^int^ cells. (B) Mice were infected with 1×10^5^ LmOVA. At the indicated time points, neutrophils and inflammatory monocytes from the spleen were analyzed for the expression of CD39 and CD73 by flow cytometry. MFI (mean fluorescence intensity) for CD39 and CD73 on neutrophils and inflammatory monocytes. Values give the mean ± SEM for three independently analyzed mice per time point and are representative for three independent experiments.
(PDF)

S3 Fig Accumulation of inflammatory cells in spleens of infected mice and production of TNF-α and IL-6 by wildtype and CD39^-/-^ spleen cells. Wildtype and CD39^-/-^ mice were i.v. infected with 5×10^3^ Lm. On day 2 post infection, spleen cells were isolated and the numbers of neutrophil granulocytes (A) and inflammatory monocytes (B) were determined (for the gating strategy see S2A Fig). Bars represent the mean ± SEM from 10 mice per group, pooled from two independent experiments. In both populations, the expression of IL-6 and TNF-α was directly analyzed by intracellular cytokine staining and flow cytometry. (C) Percentage of TNF-α^+^ neutrophils. (D) Percentage of IL-6^+^ inflammatory monocytes. (E) Percentage of IL-6^+^ neutrophils. Bars present the mean ± SEM of five individually analyzed mice and are representative for two independent experiments with three or five mice per group. Unpaired t test, ns p>0.05.
(PDF)
