# Supplementary material for: CD39 is upregulated during activation of mouse and human T cells and attenuates the immune response to Listeria monocytogenes
Source: PLoS One. 2018 May 9;13(5):e0197151. doi: 10.1371/journal.pone.0197151 (PMC5942830; doi:10.1371/journal.pone.0197151)
Supplement: S1 Fig — Wildtype mice were i.v. infected with 1×10^5^ LmOVA. At different time points post infection, spleen cells were analyzed by flow cytometry and changes in expression profile as well as percentages and numbers of different T-cell subsets were determined. (A) Representative dot plots for CD4 and CD8 gated cells. (B) Percentages of naive CD62L+CD44— and activated CD62L—CD44+ CD4+ and CD8+ T cells. Values give the mean ± SEM for 3 independently analyzed mice per time point and are representative for 3 independent experiments. (C, D) Numbers of total, CD62L+CD44—, and CD62L—CD44+ cells for CD39+ (C) and CD73+ (D) CD4+ and CD8+ T cells. Values give the mean ± SEM for 6 mice per group pooled from 2 independent experiments. Unpaired t test, comparison with day 0 (only shown for total T-cell populations), * p<0.05, ** p<0.01. (PDF) [file pone.0197151.s001.pdf]

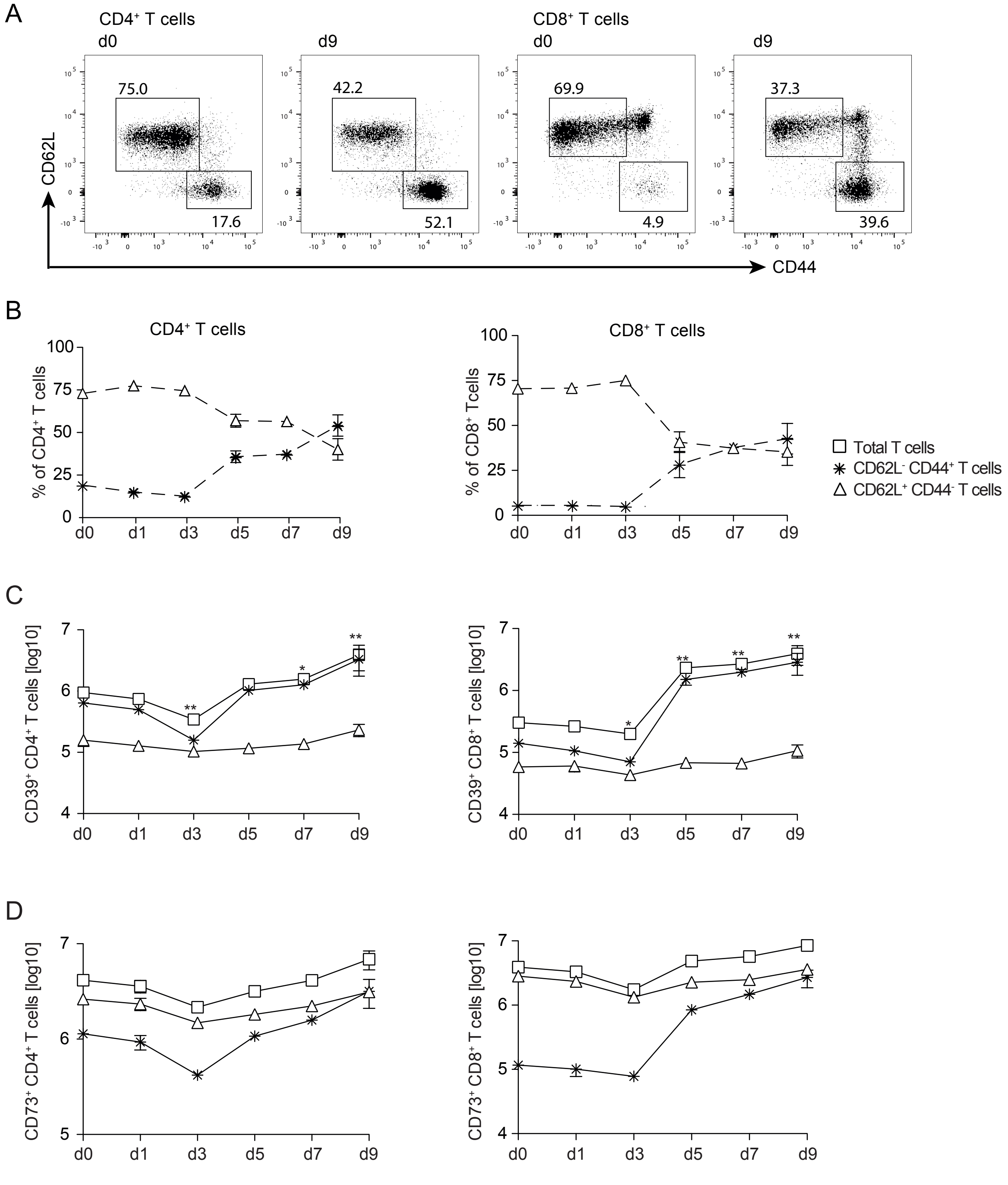

S1 Fig: CD62L and CD44 expression profiles of T cells during the course of *L. monocytogenes* infection

Wildtype mice were i.v. infected with 1×10^5 LmOVA. At different time points post infection, spleen cells were analyzed by flow cytometry and changes in expression profile as well as percentages and numbers of different T-cell subsets were determined. (A) Representative dot plots for CD4 and CD8 gated cells. (B) Percentages of naive $CD62L^+CD44^-$ and activated $CD62L^-CD44^+$ $CD4^+$ and $CD8^+$ T cells. Values give the mean ± SEM for 3 independently analyzed mice per time point and are representative for 3 independent experiments. (C, D) Numbers of total, $CD62L^+CD44^-$, and $CD62L^-CD44^+$ cells for $CD39^+$ (C) and $CD73^+$ (D) $CD4^+$ and $CD8^+$ T cells. Values give the mean ± SEM for 6 mice per group pooled from 2 independent experiments. Unpaired t test, comparison with day 0 (only shown for total T-cell populations), * $p<0.05$, ** $p<0.01$.
