# Supplementary material for: CD39 is upregulated during activation of mouse and human T cells and attenuates the immune response to Listeria monocytogenes
Source: PLoS One. 2018 May 9;13(5):e0197151. doi: 10.1371/journal.pone.0197151 (PMC5942830; doi:10.1371/journal.pone.0197151)
Supplement: S2 Fig — (A) Gating strategy: Neutrophile granulocytes were defined as CD11bhigh Ly6Cint Gr-1high and inflammatory monocytes as CD11bhigh Ly6Chigh Gr-1int cells. (B) Mice were infected with 1×10^5^ LmOVA. At the indicated time points, neutrophils and inflammatory monocytes from the spleen were analyzed for the expression of CD39 and CD73 by flow cytometry. MFI (mean fluorescence intensity) for CD39 and CD73 on neutrophils and inflammatory monocytes. Values give the mean ± SEM for three independently analyzed mice per time point and are representative for three independent experiments. (PDF) [file pone.0197151.s002.pdf]

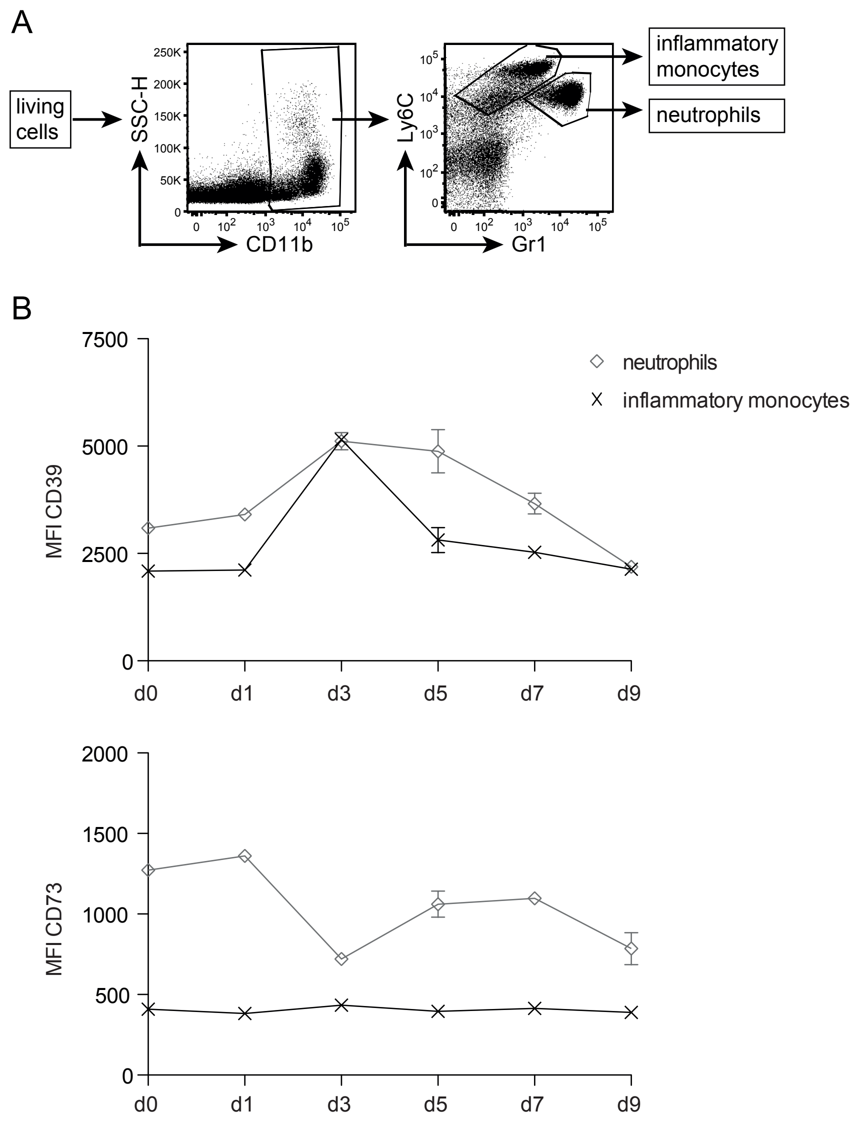

S2 Fig: Expression of CD39 and CD73 on neutrophils and inflammatory monocytes

(A) Gating strategy: Neutrophile granulocytes were defined as CD11b^{high} Ly6C^{int} Gr-1^{high} and inflammatory monocytes as CD11b^{high} Ly6C^{high} Gr-1^{int} cells. (B) Mice were infected with $1{\times}10^5$ LmOVA. At the indicated time points, neutrophils and inflammatory monocytes from the spleen were analyzed for the expression of CD39 and CD73 by flow cytometry. MFI (mean fluorescence intensity) for CD39 and CD73 on neutrophils and inflammatory monocytes. Values give the mean ± SEM for three independently analyzed mice per time point and are representative for three independent experiments.
