# Supplementary material for: CD39 is upregulated during activation of mouse and human T cells and attenuates the immune response to Listeria monocytogenes
Source: PLoS One. 2018 May 9;13(5):e0197151. doi: 10.1371/journal.pone.0197151 (PMC5942830; doi:10.1371/journal.pone.0197151)
Supplement: S3 Fig — Wildtype and CD39-/- mice were i.v. infected with 5×10^3^ Lm. On day 2 post infection, spleen cells were isolated and the numbers of neutrophil granulocytes (A) and inflammatory monocytes (B) were determined (for the gating strategy see S2A Fig). Bars represent the mean ± SEM from 10 mice per group, pooled from two independent experiments. In both populations, the expression of IL-6 and TNF-α was directly analyzed by intracellular cytokine staining and flow cytometry. (C) Percentage of TNF-α+ neutrophils. (D) Percentage of IL-6+ inflammatory monocytes. (E) Percentage of IL-6+ neutrophils. Bars present the mean ± SEM of five individually analyzed mice and are representative for two independent experiments with three or five mice per group. Unpaired t test, ns p>0.05. (PDF) [file pone.0197151.s003.pdf]

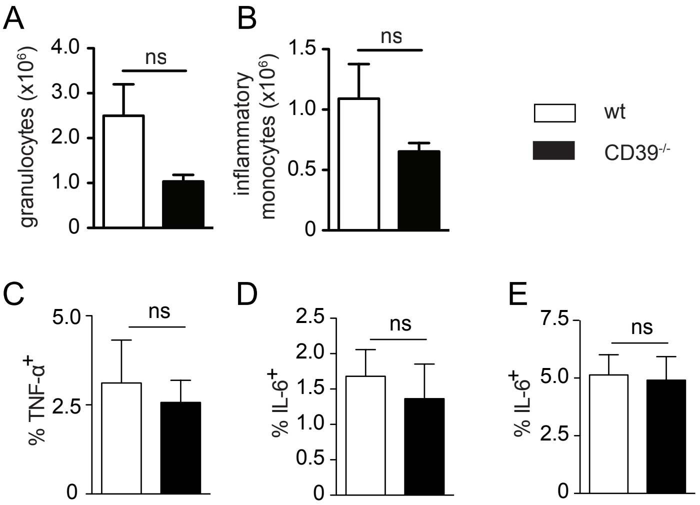

S3 Fig: Accumulation of inflammatory cells in spleens of infected mice and production of TNF-α and IL-6 by wildtype and CD39$^{-/-}$ spleen cells

Wildtype and CD39$^{-/-}$ mice were i.v. infected with 5×10^3 Lm. On day 2 post infection, spleen cells were isolated and the numbers of neutrophil granulocytes (A) and inflammatory monocytes (B) were determined (for the gating strategy see S2A Fig). Bars represent the mean ± SEM from 10 mice per group, pooled from two independent experiments. In both populations, the expression of IL-6 and TNF-α was directly analyzed by intracellular cytokine staining and flow cytometry. (C) Percentage of TNF-α^+ neutrophils. (D) Percentage of IL-6^+ inflammatory monocytes. (E) Percentage of IL-6^+ neutrophils. Bars present the mean ± SEM of five individually analyzed mice and are representative for two independent experiments with three or five mice per group. Unpaired t test, ns p>0.05.
